# A gaze-based interactive system to explore artwork imagery

**DOI:** 10.1007/s12193-021-00373-z

**Published:** 2021-05-21

**Authors:** Piercarlo Dondi, Marco Porta, Angelo Donvito, Giovanni Volpe

**Affiliations:** 1 Department of Electrical, Computer and Biomedical Engineering, University of Pavia, Via Ferrata 5, 27100 Pavia, Italy; 2 My Smart s.r.l., Via delle Officine s.n., 75100, Matera Italy; 3 COMITES srl, via Cifra 29, 20159 Milano, Italy

**Keywords:** Eye tracking, Gaze interaction, Digital humanities, Paintings

## Abstract

Interactive and immersive technologies can significantly enhance the fruition of museums and exhibits. Several studies have proved that multimedia installations can attract visitors, presenting cultural and scientific information in an appealing way. In this article, we present our workflow for achieving a gaze-based interaction with artwork imagery. We designed both a tool for creating interactive “gaze-aware” images and an eye tracking application conceived to interact with those images with the gaze. Users can display different pictures, perform pan and zoom operations, and search for regions of interest with associated multimedia content (text, image, audio, or video). Besides being an assistive technology for motor impaired people (like most gaze-based interaction applications), our solution can also be a valid alternative to the common touch screen panels present in museums, in accordance with the new safety guidelines imposed by the COVID-19 pandemic. Experiments carried out with a panel of volunteer testers have shown that the tool is usable, effective, and easy to learn.

## Introduction

The first studies on computing applied to Cultural Heritage date back to some pioneering experiments in the early ’60s. However, it is only in the ’90s that computer science, and in particular computer graphics, became able to produce significant results, from the digitization of artworks to the implementation of applications able to help archaeologists and restorers in their work [1, 2].

With the introduction of more and more sophisticated simulations and interaction modes, computer scientists became also able to support cultural and scientific dissemination. Nowadays, the adoption of interactive and immersive technologies is becoming a common approach to enhance the fruition of museums and exhibits. Famous museums all around the world (such as the Smithsonian Institution in Washington, DC, USA) now regularly exploit digital installations inside their exhibitions to present cultural and scientific information in a more appealing way. The potential of augmented and virtual reality technologies applied to Cultural Heritage has been widely studied in the scientific literature [3–5], as well as the use of serious gaming [6], particularly effective for teaching young people.

A well-designed digital installation for museums and exhibits should attract the attention of visitors, but also guarantee an easy and intuitive interaction. Gestural communication is commonly used for this purpose. In particular, Microsoft Kinect-based applications are probably the most widespread within museums [7–9], since the device is not expensive, and its basic interaction mode is potentially already known by some visitors (especially if young). However, gestural interaction is not the only viable solution for engaging visitors. Gaze interaction has a great potential in this context: being an unusual way for interacting with artworks, it can attract visitors and make them curious. Moreover, eye tracking technology is a well-known way to allow motor impaired people to communicate without using the hands, thus significantly improving the accessibility of an exhibit. Finally, the safety measures recently adopted to limit the COVID-19 pandemic advise to avoid contact with surfaces in public areas, and an eye tracking system can certainly be a safe alternative to traditional informative touch screen displays within museums.

In 2015, our research group developed a gaze-based application that allowed to interact with the images of seven tapestries depicting the famous Battle of Pavia, fought in 1525 [10]. The application was installed on three workstations available at the exhibition “1525–2015. Pavia, the Battle, the Future. Nothing was the same again”, a side event of Milan EXPO 2015, held at the Visconti Castle of Pavia (Italy) from June 13th to November 29th, 2015. The success of this event (more than 2000 visitors tried the eye tracking system) supported our hypothesis that gaze interaction can be suitable for museums and exhibits and led us to the development of the new solution we present in this paper.

Our goal is the creation of a flexible system that can be installed in any museum or exhibition to present the exposed collections in a new and interactive way. We have defined a complete workflow that goes from the choice, by experts (e.g., the curators of an exhibit or art experts), of the information to be displayed, to the interactive application that will be used by visitors. The informal feedback obtained from our first experience at the Visconti Castle exhibition has helped us to revise the initially conceived system to make it more intuitive and usable. The possibility to trigger multimedia content related to specific parts of the artwork pictures has been added too.

The article is structured as follows. Section 2 provides a brief introduction to eye tracking technology. Section 3 gives an overview of current eye tracking applications in the field of Cultural Heritage. Section 4 summarizes the main characteristics of the Visconti Castle application. Section 5 describes the proposed system and the related workflow. Section 6 presents the results of a user study conducted on two panels of volunteer participants. Finally, Sect. 7 draws some conclusions and proposes next research steps.

## Eye tracking technology

Eye movements are characterized by successions of very fast *saccades* (quick eye shifts lasting less than 100 ms) and relatively steady periods of *fixations* (with duration between 100 and 600 ms). *Eye tracking* is a general term that indicates the ability of devices called *eye trackers* to detect a person’s gaze direction [11, 12]. Practically, an eye tracker recognizes where the user is looking at and records the associated gaze coordinates, along with possible other data such as pupil size and blink rate. Visual stimuli are normally displayed on a screen, but, in principle, any visual scene in front of the user could be considered. From gaze samples (whose number depends on the eye tracker’s frequency—e.g., 60 samples per second), fixations and saccades can be derived. Most current eye trackers look like small “bars” that, placed at the base of ordinary monitors, unobtrusively record the user’s gaze.

Various methods have been designed for assessing eye movements, which differ for the technology employed. The most used techniques exploit corneal and pupillary reflections generated by infrared (or near infrared) light, that is not disturbing for the user and allows effective gaze detection in different illumination conditions.

Eye tracking technology has been exploited in several fields, such as psychology [13], neuroscience [14], marketing [15], education [16], sport [17], and usability studies [18]. In these contexts, eye tracking is usually employed to get the user’s gaze path when observing something (e.g., an image or a web page), or to obtain indications about the screen areas that were mostly observed. Interesting results have been recently achieved with wearable eye trackers, that allow users’ gaze behavior to be analyzed outside laboratories [19] and virtual reality simulations to be improved [20].

A big problem affecting the eye tracking market until a few years ago was the very high price of available devices (generally above USD 15,000), which limited their use to research purposes. However, things have now significantly changed, and some eye trackers can be bought for even less than USD 300. While their precision is sometimes a bit lower than that of expensive tools, their low price makes them potentially usable for a variety of applications, including affordable interactive installations inside museums.

When an eye tracker is exploited as an input device, gaze data need to be analyzed in real time, so that the computer can perform specific actions in response to certain gaze behaviors [12]. As said, gaze input is especially valuable as an assistive technology, for people who cannot use their hands. To date, several assistive applications have been developed, for instance for writing [21, 22], web browsing [23, 24], playing music [25, 26], or controlling videogames [27].

Pointing with the gaze is much faster than traditional solutions (like the mouse), but it also poses some challenges that need to be considered [28, 29]. Firstly, the accuracy of remote (i.e., non-wearable) eye trackers is not high—normally, approximately one degree at a distance of about 50–60 cm from the device. Consequently, relatively big interface elements are essential for comfortable interaction. Also, a way to trigger activatable elements, such as buttons or links, is needed. The “dwell time” principle is probably the most exploited, which requires the user to look at an element for a certain time for it to be triggered. The so-called Midas touch problem must be considered as well, as the primary task of gaze is normally to inspect a visual scene rather than interacting with it. Proper strategies are then necessary to distinguish visual exploration from intentional input.

Despite the above challenges, gaze-based input is vital for severely motor impaired people and can be useful to enhance the general user’s experience when interacting with multimedia content.

## Eye tracking in the cultural heritage field

Traditionally, eye tracking technology in the Cultural Heritage field has been mainly used for studying visitors’ behavior in museums [30] and their cognitive processes while observing artworks [31].

Notable works include the 2002 installation at the National Gallery of London of an autonomous public eye tracker that collected data from over 5000 visitors looking at the exposed artifacts [32, 33]; an analysis of how the context can change the perception and evaluation of modern and graffiti art [34]; and various psychological studies, for example related to emotional reactions while observing museum items [35] or to the way art paintings are observed and examined by visitors [36]. Recently, some museums, like the Cleveland Museum of Art in the United States,^1^ the ARoSArt Museum in Denmark,^2^ and the M-Museum Leuven in Belgium,^3^ have employed eye tracking installations not only to collect users’ behavior data, but also to “engage” visitors by showing them how they observed the artworks and how their gaze pattern was similar to or different from that of the other visitors.

A more interactive use of eye tracking technology involves the design of a virtual gallery that visitors can navigate using their gaze. The user can “move” inside the gallery by simply looking at the left and the right part of the screen, and then observe the chosen text, image or video [37].

The introduction of mobile eye trackers has surely helped researchers working in this field, especially for tracking how visitors observe and interact with items in museums and art galleries [31, 38, 39] or how children learn [40]. Another use of mobile eye trackers is for the implementation of museum visitor guides. A first experiment of this kind was conducted in 2011, with a prototype mobile eye tracker that acted as an unobtrusive personal guide and provided audio information on specific art objects via earphones [41]. A more recent study, conducted in 2018, compared eye-tracking-based and conventional museum guides at the Hecht Museum in Haifa, Israel [42]. In another work, related audio clips were played when specific elements in a painting were looked at by visitors wearing a mobile eye tracker [43].

Other interesting eye tracking studies in the Cultural Heritage field have investigated an author’s or art movement’s artistic and compositional techniques. Examples include the study of the effect on aesthetic perception of certain details in Chinese paintings [44], an analysis of Rembrandt’s technique which proved that the painter was able to engage the observers and direct their gaze [45], and a study about expert luthiers’ gaze behavior when trying to recognize period and author of a historical violin [46]. An overview of eye tracking applications in contemporary and new media arts can be found in [47].

## The Visconti castle gaze-based interaction system

**Fig. 1:**
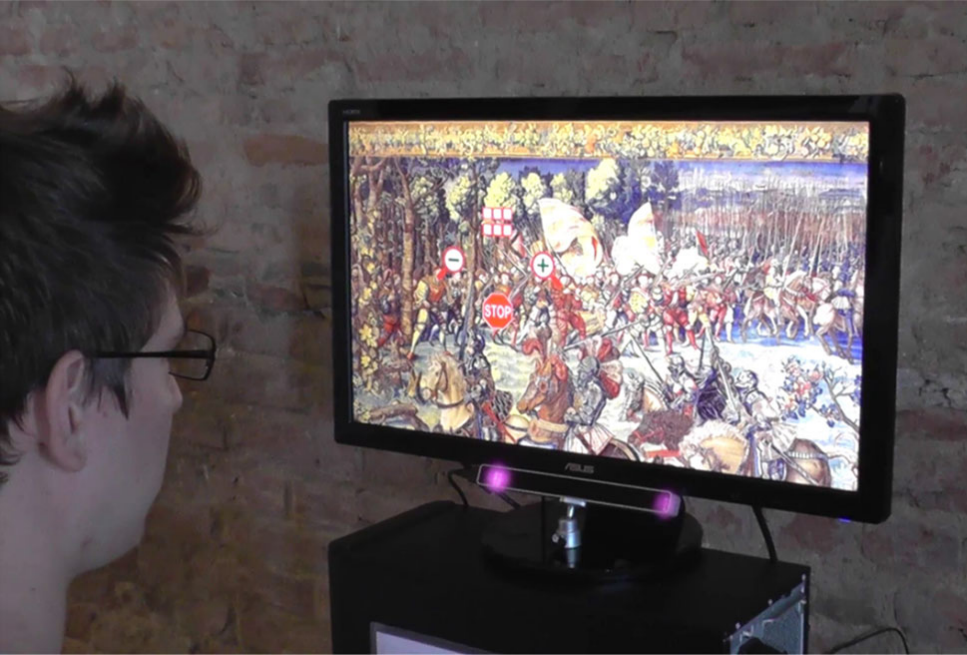
A visitor of the 2015 exhibition using the developed eye tracking application [10]

The eye tracking tool originally implemented for the “Battle of Pavia” exhibition (Fig. 1) allowed visitors to enlarge and reduce pictures of seven famous tapestries depicting scenes from the battle, perform scroll operations, and read descriptions associated with specific elements [10]. An *Eye Tribe* eye tracker was employed, a low-cost device with a sampling rate of 30 Hz.

**Fig. 2:**
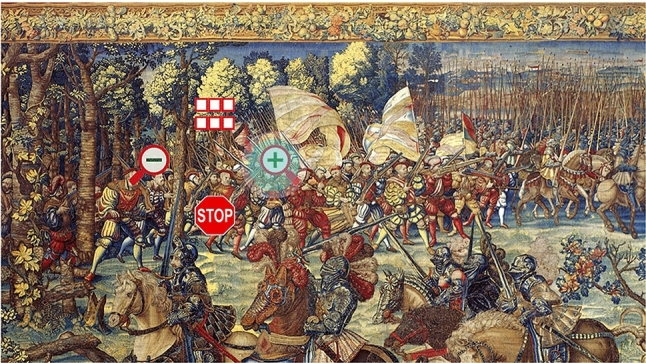
Graphical menu displayed over the tapestry picture in the 2015 application [10]

After a short calibration procedure enabling the eye tracker to find correspondences between gaze directions and specific points on the screen, the visitor was guided by a tutorial through a training session explaining how to interact with the system through the gaze. The images could then be freely surfed, zoomed, and scrolled, without time limits. Scrolling occurred by looking at the screen edges. Other actions could be performed through a graphical menu displayed when any point of the image was fixated for a specific time. The menu (Fig. 2) included icons allowing to trigger zoom in, zoom out, and new picture selection operations, as well as to stop the interaction process. The pictures contained “sensitive areas”, i.e., regions that, when observed, were highlighted with a semi-transparent yellow rectangle. A short descriptive text was displayed close to these areas, to provide the visitor with contextual information.

Three eye tracking devices were available at the exhibition. More than 2000 visitors tried the eye tracking system, at least partially. Despite some problems due to the limited calibration performance of the employed (cheap) eye tracker, this event confirmed our idea that eye tracking technology can potentially enhance the visitor’s experience at an exhibition, besides being (inherently) very important for accessibility. The users’ feedback collected during the event also helped us to identify problems and limitations of the system, providing precious hints on how to improve it. More precisely, the most recurrent concerns raised by visitors were: (i) the excessive length of the interactive tutorial; (ii) the *stop* and *menu* buttons placed too close to the zoom in/out controls; (iii) unwanted scrolling when observing the edges of the image. We also noticed that only few visitors found all the active areas inside each image. Each issue and the corresponding proposed solution will be discussed in Sect. 5.2.

## The proposed solution

**Fig. 3:**
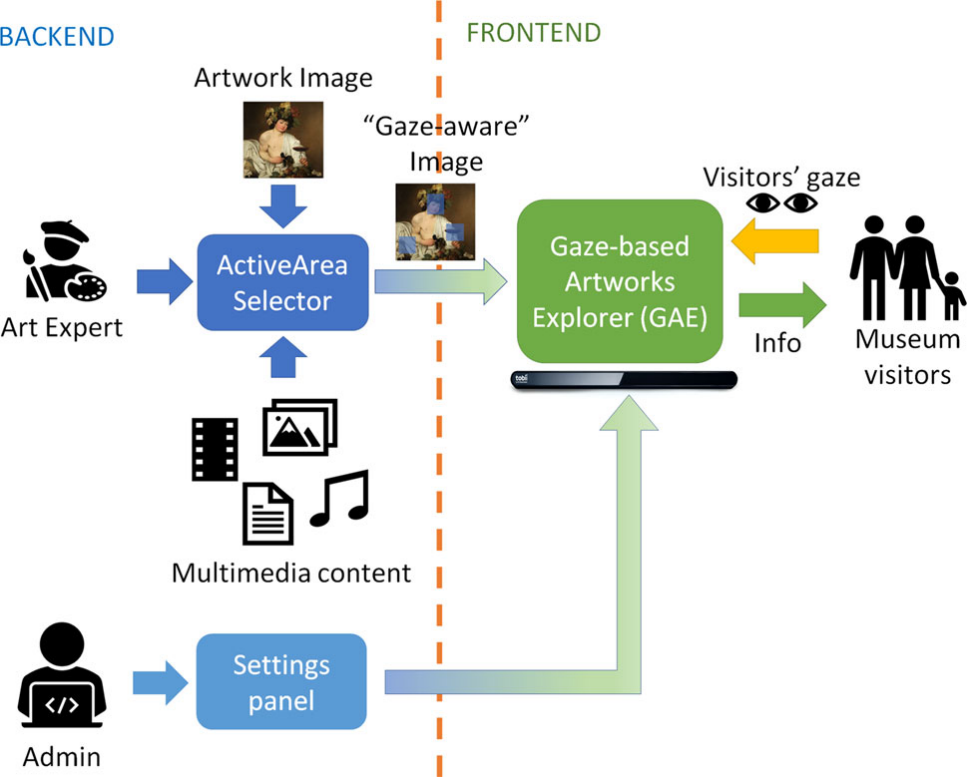
High-level workflow of the proposed system

Our system allows to implement an effective workflow for gaze-based interaction with artwork imagery and is composed of two main parts (Fig. 3):

- a *backend*, consisting of two tools, one to be used by art experts (e.g., the curators of an exhibition) to define the contents that will be displayed, and the other by the system administrator, to choose the various settings;
- a *frontend*, consisting of a single application, that is the actual eye tracking interface used by end users (i.e., visitors).

### Backend

The first backend tool, called *ActiveArea Selector*, allows to easily create “gaze-aware” images and define and update the contents that will be shown in the frontend application. In a museum, the set-up of the exhibit often changes when new artworks arrive on loan for a short period, or for important anniversaries and events. In all these cases, also the multimedia content of an interactive installation needs to be updated accordingly. To make this operation as simple as possible, an art expert can use the *ActiveArea Selector* tool to load an artwork image, “draw” rectangular “regions of interest” (*active areas*) on it, and link specific multimedia content (text, image, audio, video, or their combination) to each of them. Some multimedia contents can only be played separately. For example, while it is possible to combine a caption, an audio clip, and an image, it is not possible to play audio and video clips at the same time, or to show an image and a video together—which would be rather confusing for the visitor. The chosen contents can enhance the fruition of the artwork, supplying additional historical, artistic, or scientific information.

**Fig. 4:**
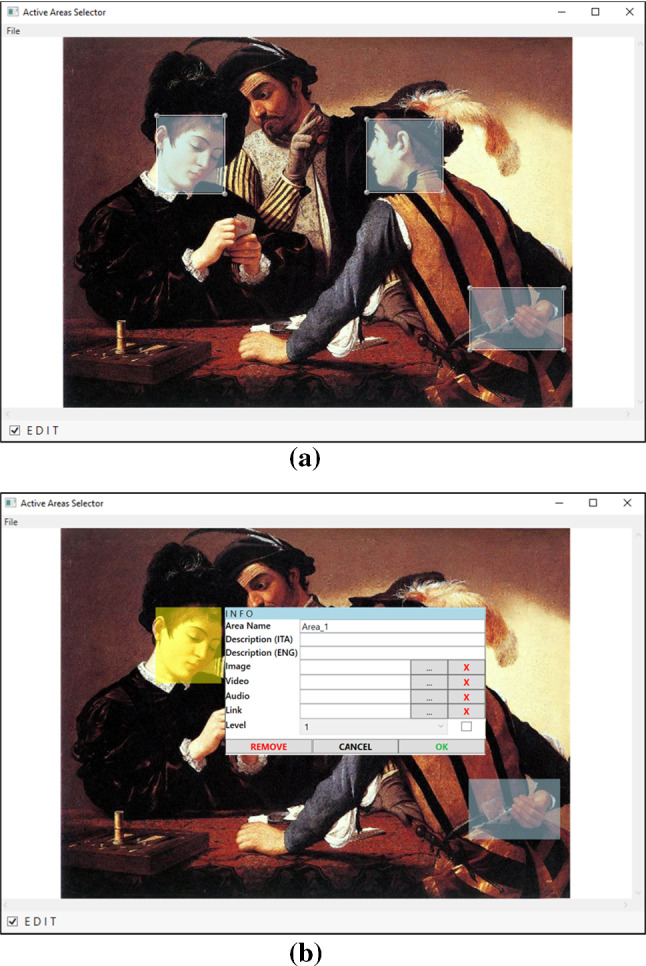
Graphical user interface of the *ActiveArea Selector* tool (the sample image is *The Cardsharps* painted by Caravaggio in 1594): **a** three active areas drawn on the image (highlighted in light blue) and **b** content menu of a selected active area (highlighted in yellow)

The graphical user interface of the *ActiveArea Selector* is minimal, to be easily comprehensible to users who may be expert in art but not necessarily in technology (Fig. 4). The main panel is occupied by the image, that can be panned and zoomed using the lateral scroll bars and the mouse wheel. By activating the “edit mode”, through a checkbox at the bottom of the screen, the mouse can be used to draw, move, and re-scale active areas (Fig. 4a). A right click on an active area displays a pop-up menu (Fig. 4b) that allows to enter a description, in different languages and choose or update the linked media content. The selected active area changes its color from light blue to yellow to differentiate itself from the other areas and provide a visual feedback. Once all areas have been drawn and their content has been added, a “gaze-aware” image, coded through an XML file, is exported for the frontend application.

Even if there is no limit to the number of active areas that can be defined for an image, it would be better not to have too many of these sensitive regions, to avoid cognitively “overloading” the visitor with too much information and multimedia content. Basically, only some meaningful features of the artwork should be highlighted, that can be easily understood by visitors in a short time. After some trials during the development of the system, we found that up to three active areas are suitable for “small” images, while more areas (five or six) can be employed for bigger pictures. However, the tool also offers an additional option in case of images with a large number of active areas (e.g., a painting with many characters). Active areas can be grouped into “levels”, so that the user can selectively display the areas of a specific level only. This occurs with an additional selection menu that appears when a picture with associated levels is opened (see Sect. 5.2). This option should only be used for “complex” paintings, with many relevant elements.

To speed up the initial image annotation phase, the *ActiveArea Selector* tool can be used by different art experts in parallel, each working on a different artwork.

The second backend tool is a settings panel used by the system administrator to set all the parameters of the frontend application, such as the colors of graphical elements, timers, maximum levels of image zoom, or the behavior of the active areas. In this case too, we opted for a simple interface showing, for each parameter, its current value and a short description explaining the valid values. Also, this tool does not require specific technical knowledge and can be effectively used by the curator of an exhibition to customize the user experience.

### Frontend: *Gaze-based Artwork Explorer* (GAE)

**Fig. 5:**
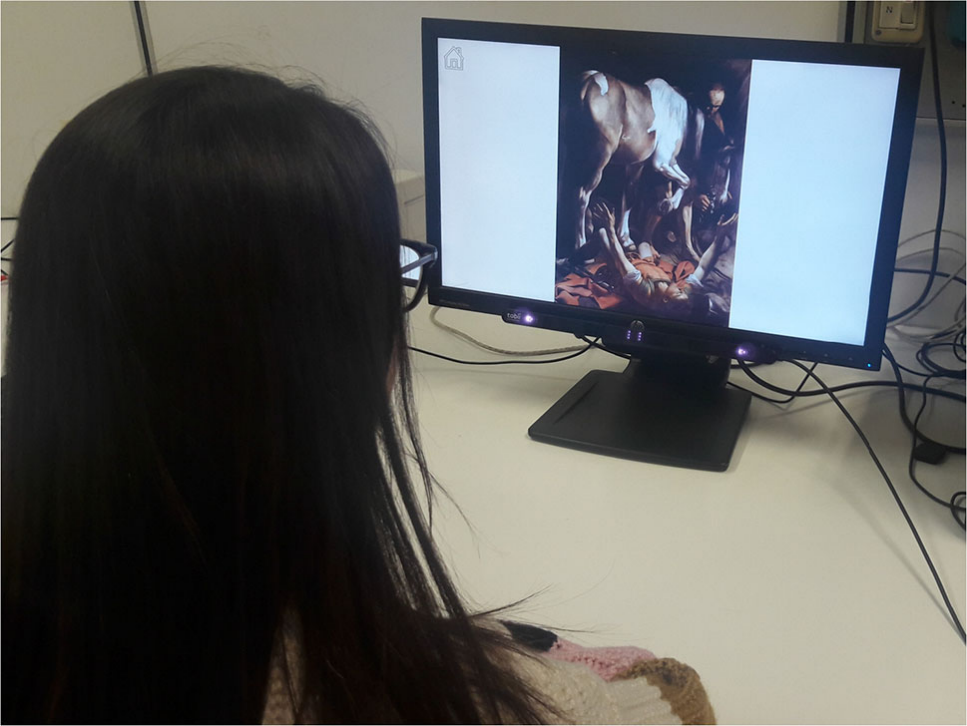
A user trying the *GAE* tool in a laboratory setting

The frontend is the core of the proposed system. The setup consists of the eye tracker placed at the base of a computer screen (Fig. 5). The user sits in front of it and, after a short calibration procedure (that simply consists in looking at a few circles displayed in different positions), can interact with the application. Instead of the Eye Tribe eye tracker employed in the previous exhibition, now out of production, we have used the more recent *Tobii 4C*, together with its SDK that integrates well with the Microsoft C# Windows Presentation Foundation (WFP)—the standard framework for creating user interfaces in Windows-based applications.

**Fig. 6:**
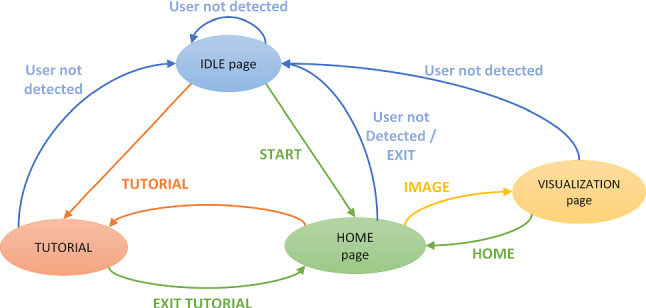
State diagram of the *GAE* tool. A transition to a different state is triggered by events (e.g., when clicking a button or completing the tutorial)

**Fig. 7:**
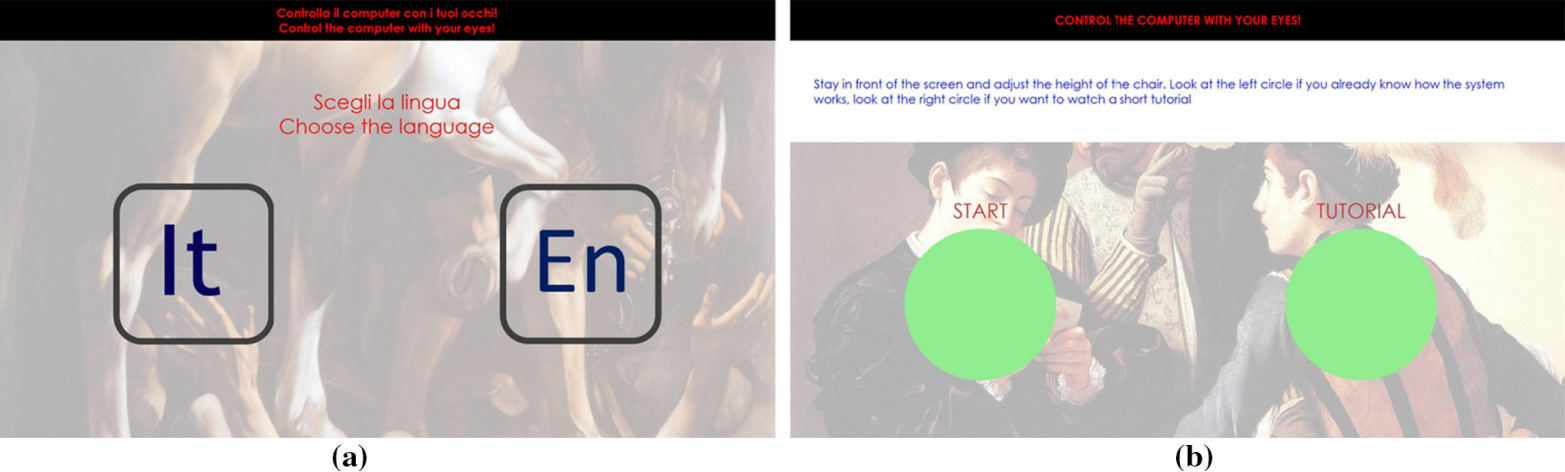
*Idle* page: **a** language selection; **b** start/tutorial choice

The development of the eye tracking tool, called *Gaze-based Artworks Explorer* (shortened to *GAE* from now on), followed three main design principles: intuitiveness, generality, and robustness.

Both the interface and the interaction were designed to be as intuitive as possible: a multimedia application in a museum is generally used only one time, and thus it must be immediately comprehensible to the user and be characterized by a very quick learning curve. *GAE* supports different kinds of media, and its “behaviors” (such as the duration of a *dwell time*, i.e., the fixation duration for an action to be triggered) can be customized by the administrator. Finally, to make the system robust, all buttons are large and well-spaced, so as to compensate for possible errors due, for example, to big movements of the user in front of the screen or to sub-optimal calibrations—both possible in a crowded setting like a museum.

Figure 6 shows the system’s state diagram, illustrating how a user can move among the various “pages”.

The starting point is the *Idle* page (Fig. 7), that allows the user to choose the language and then to reach the *Home* page or to run a tutorial. The graphics in this page is very simple, to be easily understood by users who, at this step, do not know how to use the system yet. Every button in the application changes its color when the user’s gaze is perceived on it, to provide a visual feedback. A button is considered “pressed” after a certain dwell time (1 s as a default). This prevents accidental “clicks” by inexpert users who have never used an eye-controlled interface before. All the default values (such as dwell times, highlight colors, or zoom and scroll speeds) can be changed in the system’s settings. Different behaviors can be set for different controls—for example, a short dwell time for the *start* button and a longer time for the *exit* button.

**Fig. 8:**
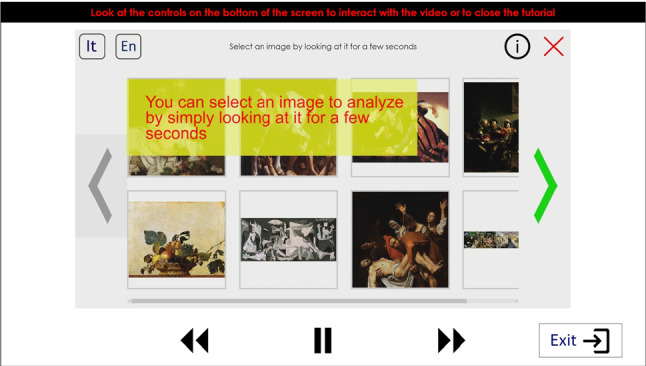
*Tutorial* page

The *Tutorial* page (Fig. 8) shows a short video (about 90 s) explaining how to interact with the tool. The user can stop, move forward, or move backward the video with controls available at the bottom of the page. When the video ends, or by selecting the *exit* button, the Home page is loaded. This is a simplification with respect to our initial implementation, in which, during the tutorial, the user had to actively try each control. We adopted this strategy because the interactive tutorial was judged too long and annoying by the visitors of the “Battle of Pavia” exhibition. We also noticed that some of them stopped using the application a few minutes after the end of the tutorial. Even if, in general, an interactive tutorial is an effective solution to learn a new software, normally visitors of a museum do not want to learn how to use an application: they only desire to quickly obtain information about the exposed artworks. We thus shortened the tutorial and uniformed the interaction mode to make the application easier and quicker to learn. This is the reason why all buttons have the same behavior and the same visual feedback, and why we used only standard and intuitive icons (such as arrows or magnifying lenses).

**Fig. 9:**
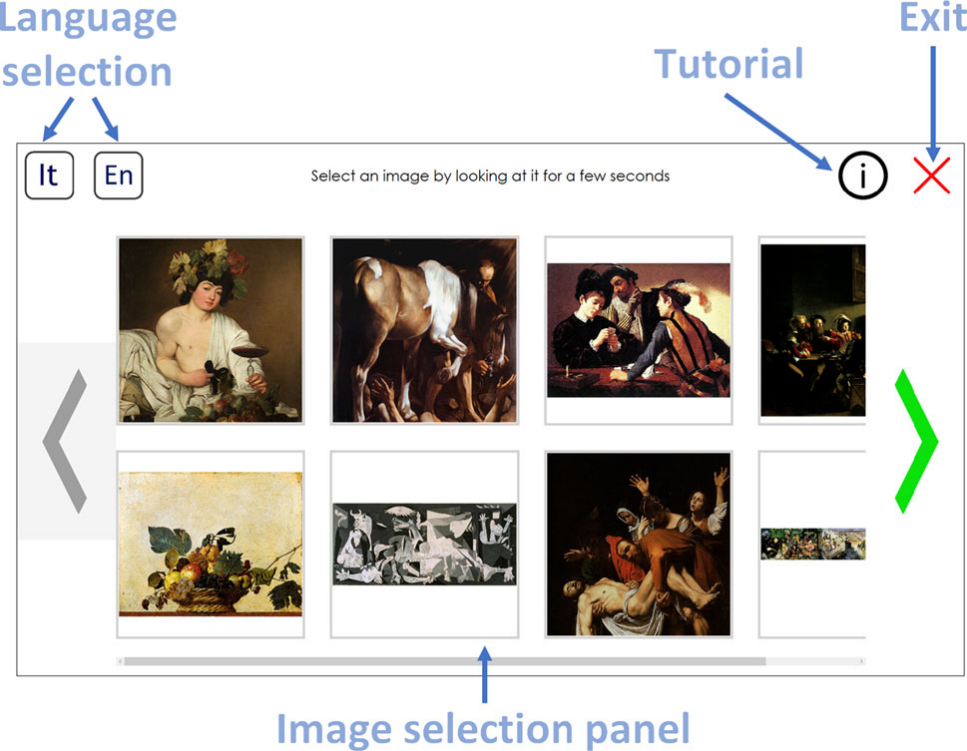
*Home* page showing the available paintings and control buttons

The *Home* page (Fig. 9) acts as a control panel for the user, who can choose which artwork to explore among those available, change the current language, run the tutorial again, or exit. Artwork images are shown as thumbnails that can be selected like buttons. Arrows at the left and right sides of the screen allow to horizontally scroll the thumbnails, when necessary.

**Fig. 10:**
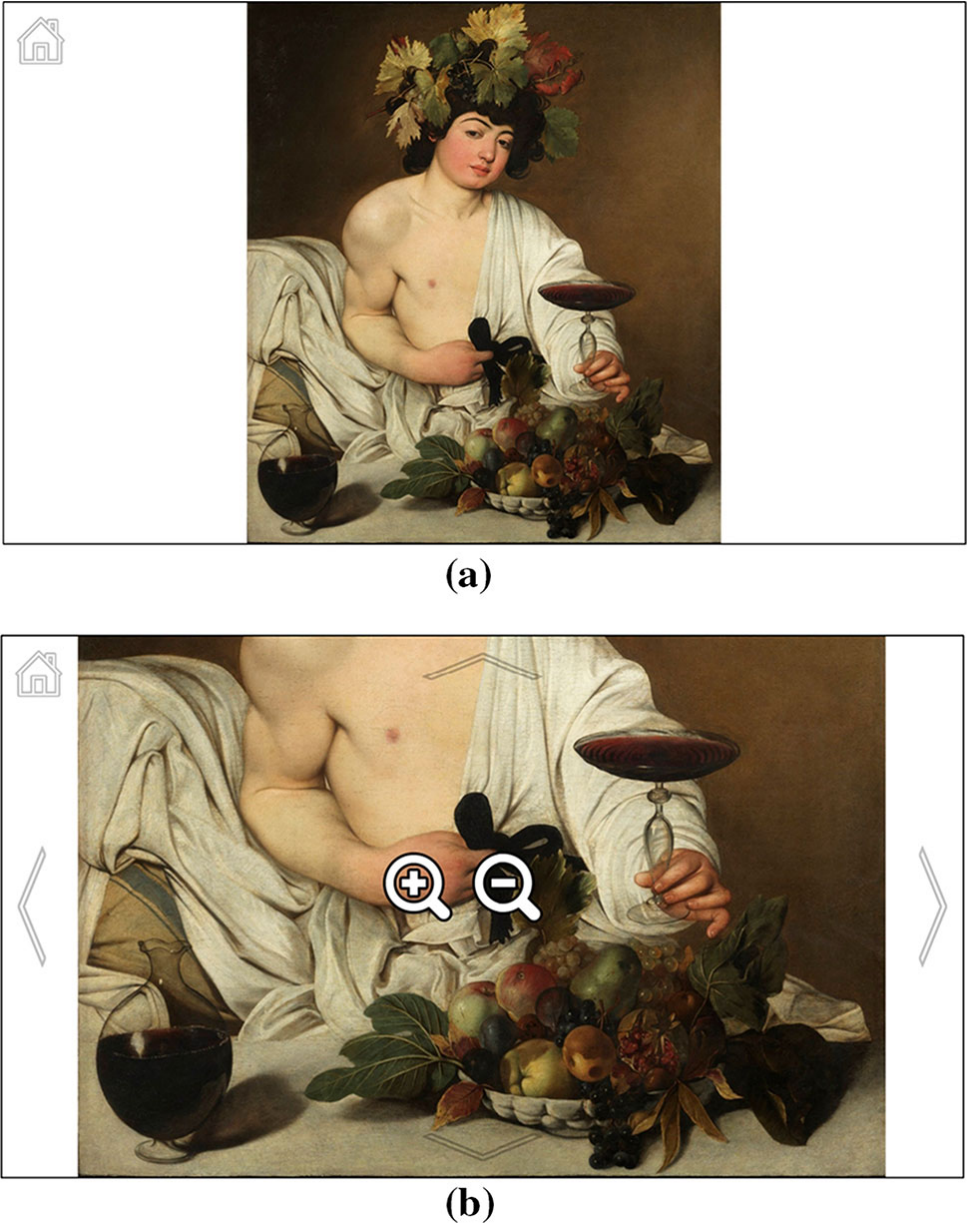
*Visualization* page: **a** painting when initially loaded (*Bacchus* by Caravaggio, 1595); **b** painting after pan and zoom operations

The *Visualization* page (Fig. 10) is the core of *GAE*. Its graphic structure and interaction mode have been completely redesigned with respect to the Visconti Castle application. In that case, all buttons were hidden and a visual menu (allowing to zoom in/out, change the current image, and exit the program) appeared when any area of the picture was fixated for 2 s (Fig. 2). This approach was deemed not completely comfortable by visitors, since zoom in/out operations are much more frequent than changing the displayed picture or abandoning the application. In general, the presence of unnecessary functions may confuse the user and potentially lead to wrong actions. In *GAE* we have logically separated the navigation within an image from the navigation within the application.

**Fig. 11:**
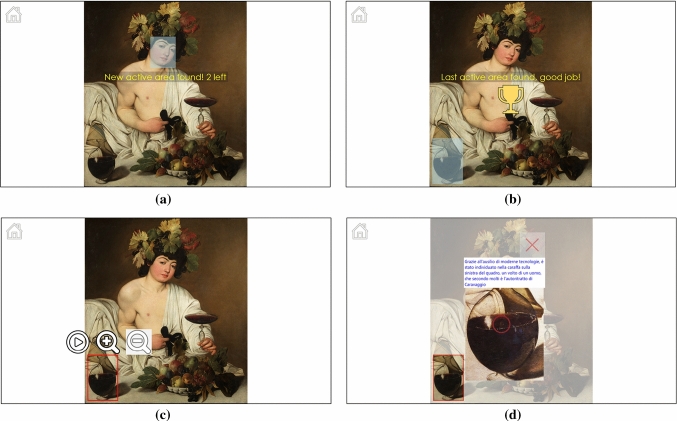
Active areas within a painting: **a** an active area has just been found and is highlighted; **b** the last active area in the painting has been found; **c** graphical menu of an active area; **d** media content associated with an active area

A *home* button is always visible in the upper left corner, allowing to return to the *Home* page. By fixating any spot of the image for a certain time (2 s by default), two *lens* buttons appear through which it is possible to perform zoom in and out operations on the observed area. By looking at any other part of the image (for half a second by default), the lenses disappear. When the image is zoomed in, four arrow buttons appear at the four edges of the screen allowing to scroll the image (Fig. 10b). Each button is semi-transparent, but it changes its color to light blue when the gaze is detected on it. This is an acceptable compromise between making the buttons well visible and hiding the image as less as possible. This choice also solves a problem of the previous application, in which the visitor could perform scrolls by looking at any parts of the screen’s edges. Even if that solution was reasonable and intuitive, we noticed that it could cause accidental scrolls when the user was simply observing the edges of a painting. Having explicit buttons makes the user more aware of the position of controls, thus reducing possible accidental shifts of the image. To avoid abrupt movements, both zoom and scroll operations are initially slow and increase their speed only if the user’s gaze remains on the buttons.

The displayed image can contain active areas previously defined with the *ActiveArea Selector* tool (Sect. 5.1). Active areas are not visible at the beginning. Only when the user’s gaze is over one of them, its rectangular region is highlighted in light blue (Fig. 11a, b). Active areas were also present in the Visconti Castle application, but, in that case, they could only provide textual information, such as the name of a depicted character. No initial information about the number of available active areas was provided. However, at the exhibition we noticed that only few visitors actually found all the active areas: after the first ones, their interest seemed to diminish. To potentially engage visitors more, we have introduced some gamification principles, adding a sort of “reward” when all the areas are found. As soon as a new image is loaded, a message displayed at the center of the screen informs the user about the number of active areas present in that picture and invites him or her to find all of them. When the user’s gaze is detected for the first time on an active area, a “congratulation message” appears which informs about the number of remaining areas (Fig. 11a). When all the areas have been found, a “cup” is shown (Fig. 11b). This sort of simple mini-game can encourage visitors to explore the entire artwork and find all the available active areas with the associated media content.

**Fig. 12:**
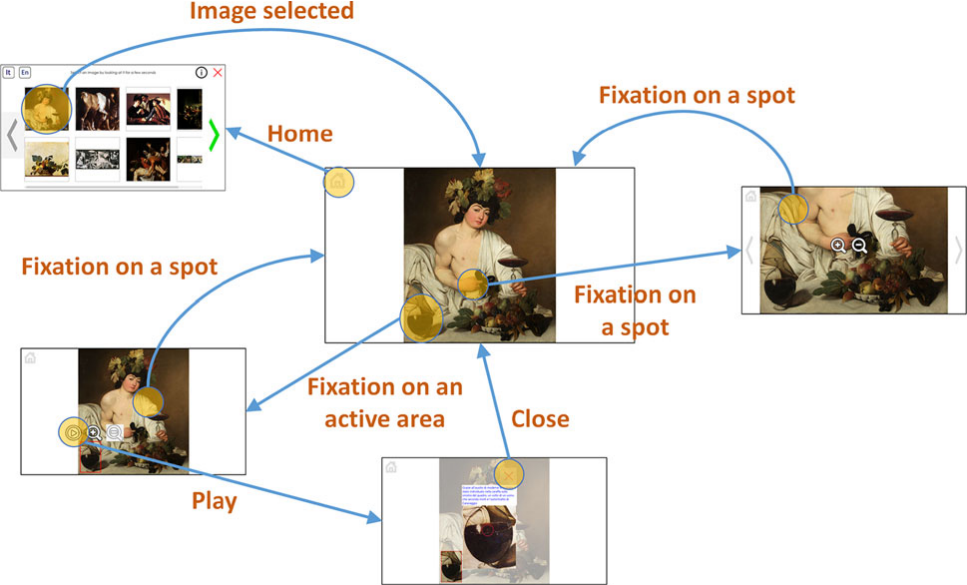
Example of interaction in the *Visualization* page (fixations are indicated with yellow circles)

When an active area is fixated, a *play* button appears besides the two zoom lenses (Fig. 11c). The area is then highlighted with a thin red border, with no background color. Actually, when the user is searching for active areas, a well-evident blue background is better so as to make the areas evident; on the contrary, when the user focuses his or her attention on a specific region, it is better to show the original colors of the painting. Looking at the *play* button triggers the display of the associated multimedia content, shown in a pop-up panel (Fig. 11d). The appearance of this panel automatically hides all buttons and makes the whole image semi-transparent except for the active area. This way, the attention of the user can be focused on the media content and on the related active area. To close the pop-up panel, the user has to simply look at the *X* button. Optionally, the media content can be directly opened as soon as the active area is fixated for a predefined time. It is important to stress that, in both cases, the activation of the multimedia content is consciously triggered by the user (with a long fixation or the “press” of the the *play* button). Thus, if the visitor is not interested or has already explored all the multimedia elements, s/he can simply ignore them and continue looking at the painting without distractions. Figure 12 shows an example of interaction in the *Visualization* page.

**Fig. 13:**
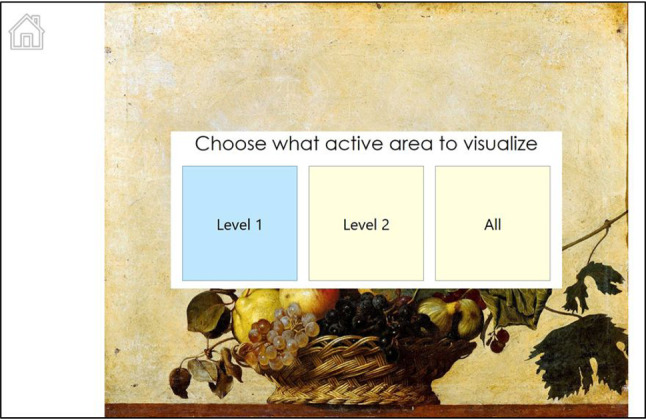
Menu for the selection of the group of active areas

As stated in Sect. 5.1, when there are many active areas in an image, they can be grouped into “levels” by the art expert and then displayed one at a time by the user. When the *Visualization* page is loaded, a visual menu appears through which the user can choose which group of active areas to show, by simply looking at the correspondent button (Fig. 13). The buttons in the menu will be as many as the number of levels previously defined (for the specific image) by the art expert using the *ActiveArea Selector* tool.

At the end of the interaction experience, the user can either exit the application selecting the corresponding button in the *Home* page or simply move away. In any page, if the user is not detected for a few seconds (10 as a default), the system resets itself and return to the *Idle* page.

## User study

In order to evaluate both the backend and frontend components of our system, the user study was divided into two parts: one is a qualitative analysis of *ActiveArea Selector*, conducted on a small group of volunteer subjects; the other is a quantitative analysis of *GAE*, conducted on a larger panel of participants.

### Backend: *ActiveArea Selector*

To evaluate the usability of the tool, we involved 7 volunteer participants (5 males and 2 females) aged between 25 and 59 (42 on average). After a short explanation of the controls and functionalities of *ActiveArea Selector*, and after 5 min of training, each participant was asked to perform the following tasks:

- Open an image.
- Repeat for three times the following steps:
  (i) Chose a spot, zoom-in and draw an active area on it.
  (ii) Add a multimedia content to the just drawn active area.
  (iii) Zoom-out and resize the active area.
- Save the project and close the program.
- Open the program and the project again.
- Choose one of the active areas and move it to a different position.
- Remove the multimedia content of the area and add a new one.
- Save the project and close the program.

The above list includes the main operations that can be performed using *ActiveArea Selector*. Each participant repeated the tasks $a-g$ for three times, each time using a different image. To simulate the actual work of the curator of an exhibition, the participants were free to choose the images from a sample set of artworks and to add the multimedia contents they preferred. Apart from some initial uncertainty (for example, in the first attempt someone did not exactly remember all the commands), no participant made mistakes.

**Table 1:** The SUS questionnaire

Statements	
(1) I think that I would like to use this system frequently	
(2) I found the system unnecessarily complex	
(3) I thought the system was easy to use	
(4) I think that I would need the support of a technical person to be able to use this system	
(5) I found the various functions in this system were well integrated	
(6) I thought there was too much inconsistency in this system	
(7) I would imagine that most people would learn to use this system very quickly	
(8) I found the system very cumbersome to use	
(9) I felt very confident using the system	
(10) I needed to learn a lot of things before I could get going with this system	

At the end of the test, the participants were asked to fill in a standard System Usability Scale (SUS) questionnaire [48]. Each of the ten statements of the survey (Table 1) was ranked with a five-level Likert scale, from 1 (“I do not agree at all”) to 5 (“I totally agree”). The SUS score, scaled between 0 and 100, was computed as follows: for the odd-numbered sentences, 1 was subtracted from the participant’s responses; for the even-numbered sentences, the participant’s responses were subtracted from 5; the results were then added together, and the obtained value was multiplied by 2.5. The obtained mean SUS score was 89.3, that is rated ‘excellent’ according to the adjective ratings proposed by Bangor et al. [49] and ‘A+’ accordingly to the grading scale proposed by Sauro and Lewis [50].

### Frontend: *GAE*

While eye tracking is a well-established assistive technology for motor impaired people, we wanted to verify whether *GAE* can be valid in general, both as an “engaging” tool to attract visitors in a museum and as an alternative to traditional information devices like touch screen displays.

During the implementation phase, we conducted several informal tests with a few users to refine *GAE* and choose the default parameters of the system. Then, we performed a formal user study on the final version of the application, with 33 volunteer participants (21 males and 12 females aged between 22 and 71) to verify its usability and effectiveness. Only seven participants had prior experience with eye tracking applications. We did not perform a longitudinal study because our target users are visitors of a museum, who will probably interact with the system only one time and briefly. Thus, it is more important to verify whether the interface is intuitive, and the interaction modes are easy to understand for new users.

**Table 2:** The proposed questionnaire for *GAE* with mean values and standard deviations (STD) of testers’ responses

Statement	Mean	STD
(1) The interface is intuitive (controls’ position, visual feedback, layout, etc.)	4.67	0.54
(2) It is easy to learn how to use the tool	4.79	0.48
(3) Gaze interaction is comfortable	4.09	0.63
(4) The tool is reactive to gaze input	4.18	0.68
(5) Completing the given task was easy	4.82	0.39
(6) I would recommend this tool to others	4.70	0.47
(7) I think that this kind of interactive presentation is effective for learning new information about artworks	4.55	0.71
(8) I think that this application fits well in museums or exhibitions	4.70	0.47

**Fig. 14:**
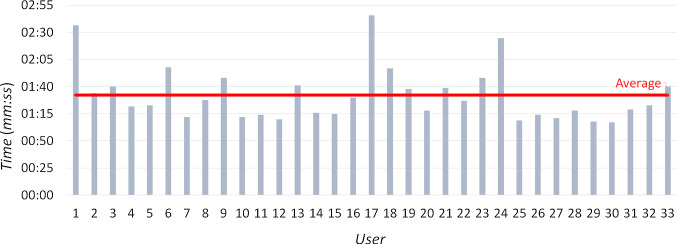
Time taken by each participant in the user study to complete the given task with *GAE*

After a brief explanation of the functioning of the system, the participants could freely use it for 2 min. Then, starting from the Home page, we asked them to perform the following operations:

- Open an image, specifically *Bacchus* by Caravaggio.
- Fully zoom-in on the right shoulder of Bacchus.
- Scroll the zoomed image to reach the right hand of Bacchus, at the center.
- Zoom-out to the original size of the image.
- Find all the active areas present in the image (three) and, for each, open and close the connected media.
- Return to the *Home* page.

The above list includes all the typical operations performed during a “standard” interaction (a quick free navigation in a museum). Considering the initial explanation time, the free use, and the actual test, each participant interacted with *GAE* for approximately 5 min.

At the end of the experiment, we asked each participant to fill in a short questionnaire (Table 2) aimed at assessing the perceived “usefulness” and effectiveness of the tool. Each statement was ranked with a five-level Likert scale, from 1 (“I do not agree at all”) to 5 (“I totally agree”). We also encouraged participants to explain the reasons for their votes. In this case, we did not use the SUS questionnaire, since some statements were not suitable (e.g., the first), and others were too generic for our case and the evaluation might have been biased by the non-standard nature of the interaction modality. In fact, for the majority of the testers this was the first time they tried gaze-based interaction, and it was thus obvious that they were not entirely confident with it. These “nuances” might have been lost using an overall usability score like SUS; we thus preferred to use an ad hoc designed questionnaire, with statements more targeted at our specific gaze-based system (e.g., “Gaze interaction is comfortable”, or “The tool is reactive to gaze input”). We tried to focus on the assessment of the peculiar features of our tool, especially the comfort of gaze interaction and its applicability in museums.

The obtained mean scores are always above 4, and even above 4.5 in six cases out of eight. Participants found the interface intuitive (statement 1) and the tool easy to learn (statement 2). Statements 3 (comfort) and 4 (reactivity) were among the most divisive. This was expected, since gaze interaction is less intuitive and slower than more common input modalities, such as those based on mouse or gestures—especially for users at their very first experience. Moreover, the perceived level of reactivity to gaze input is a very subjective matter. For example, the dwell time to display zoom in/out controls was considered too long by some participants and too short by others. Since there is no absolute solution, the default dwell time of 2 s was chosen as a reasonable compromise between sufficiently fast interaction and comfortable observation of images without the display of (possibly disturbing) pop-up elements.

Completing the assigned task was easy for participants (statement 5). This is an interesting result, since, despite some subjective preferences about dwell times or operation speeds, all participants found using the tool easy, and they would also recommend it to others (statement 6). Finally, also the effectiveness for learning achieved a high score (sentence 7), and most participants deemed the application suitable for museums or exhibitions (statement 8).

Regarding performance, all the participants succeeded in completing the assigned tasks without errors, apart from some occasional uncertainty in achieving the goals. None of them reported tiredness or fatigue. However, since *GAE* has been designed and tested for short-time use, as usually happens for multimedia applications in museums, we cannot exclude that fatigue may occur after a long time. The mean completion time was 1 min and 32 s (STD 25 s). Only three participants, who had some problems with the initial calibration due to strong corrective glasses or bluish eyes (known issues of eye tracking technology [51]), needed around 2 min and a half (Fig. 14). With a poor calibration, the eye tracker cannot perfectly track the user’s gaze, and, in the worst case, it may also “lose” one or both eyes for a short time. When this occurs, the user must repeat the intended action, which leads to a longer total execution time. Excluding these participants, the results were significantly more uniform (mean 1 min and 25 s, STD 15 s).

Overall, we can say that *GAE* proved to be robust, since even subjects with a sub-optimal calibration were able to properly use the system and only experienced some delays in the selection or activation of controls. Furthermore, an average time of about a minute and a half to explore an artwork means that the system is fast enough to allow visitors to explore multiple “gaze-aware” images during their visit to the museum in a reasonable time.

## Conclusions

In this paper we have presented a workflow for designing gaze-based interactive installations in museums and exhibits. We have created both a tool that art experts can use to easily enrich artwork pictures with multimedia content and an interactive application through which visitors can explore “augmented” images using only their gaze—which is also very important for accessibility, in case of people who have difficulties in using their hands. Experiments conducted on a panel of volunteer participants have provided more than satisfying results in terms of performance and perceived usability and effectiveness.

Several museums and art galleries are now suffering from a decrease in visitors, as well as from a lack of funds and investments. As demonstrated in scientific literature, “engaging” technological installations can be a good way to attract visitors, especially the younger generation. In a sense, appealing interaction technology may become a sort of “Trojan horse” that can help in enticing people to visit museums and exhibitions. Moreover, the current limitations imposed by the COVID-19 pandemic push to create new safe ways to interact with shared devices in public places: a gaze-based system can be a valid alternative to traditional solutions like touch screen displays.

Of course, the proposed application could also be used outside buildings or events specifically devoted to arts. Provided that the user has an eye tracker (which is now possible thanks to the strong decrease in prices of these devices), the developed tool could be employed at home as well. Even if, still in this case, the “attractive” component would play an important role for everybody, the most relevant advantage would be for severely motor-impaired people, who cannot (or for whom it would be very difficult to) physically visit a museum: in the name of the universality or art, nobody should be excluded from the fruition of cultural heritage.

Future work will likely include some technological improvements (e.g., to make the system work with different kinds of eye trackers), the addition of new functionalities to the application, and further tests (both outside and inside museums). Possible calibration-free versions of the system will be investigated, too.

## Data Availability

All images of paintings shown in this paper are in the public domain.

## Footnotes

1 https://mw18.mwconf.org/glami/gaze-tracker/.

2 https://userexperienceawards.com/2017-submissions/aros-art-museum-aros-public/.

3 https://www.mleuven.be/en/eye-tracking-research-m.

## Code availability (software application or custom code)

The code is not publicly available.
